# Advancing pediatric palliative care in a low-middle income country: an implementation study, a challenging but not impossible task

**DOI:** 10.1186/s12904-020-00674-2

**Published:** 2020-11-06

**Authors:** Ximena Garcia-Quintero, Luis Gabriel Parra-Lara, Angelica Claros-Hulbert, Maria Isabel Cuervo-Suarez, Wendy Gomez-Garcia, Francois Desbrandes, Natalia Arias-Casais

**Affiliations:** 1 Fundación Valle del Lili, Department of Pediatric Palliative Care , Cra 98 # 18 -49, Cali, 760032 Colombia; 2 Centro de Investigaciones Clínicas (CIC), Fundación Valle del Lili, Cali, 760032 Colombia; 3 Facultad de Ciencias de la Salud, Universidad Icesi, Calle 18 #, 122-135 Cali, Colombia; 4 Dr. Robert Reid Cabral Children’s Hospital, Santo Domingo, Dominican Republic; 5 Pediatric Oncology My Child Matters Program, Pediatric Oncology, Lyon, France; 6 ATLANTES Global Observatory for Palliative Care, Instituto Cultura y Sociedad (ICS), Universidad de Navarra, Pamplona, Spain

**Keywords:** Pediatric, Pediatric palliative care, Program, Palliative medicine, Implementation, Terminal care, Latin America

## Abstract

**Background:**

The disparities in access to pediatric palliative care and pain management in Latin America remains an unaddressed global health issue. Efforts to improve the development of Palliative Care (PC) provision have traditionally targeted services for adults, leaving the pediatric population unaddressed. Examples of such services are scarce and should be portrayed in scientific literature to inform decision-makers and service providers on models of care available to tackle the burden of Pediatric Palliative Care (PPC) in Low-and middle-income countries (LMIC). The purpose of this study is to describe the implementation of a pediatric palliative care program, “Taking Care of You*” (TCY)*, in a tertiary care, university hospital in Cali, Colombia.

**Methods:**

A program’s database was built with children between 0 to 18 years old and their families, from year 2017 to 2019. Descriptive analysis was carried out to evaluate the impact of the program and service delivery. A theory-based method was directed to describe the PPC program, according to the implementation of self-designed taxonomy, mapping theoretical levels and domains. Clinical outcomes in patients were included in the analysis.

**Results:**

Since 2017 the program has provided PPC services to 1.965 children. Most of them had an oncologic diagnosis and were referred from hospitalization services (53%). The number of ambulatory patients increased by 80% every trimester between 2017 and 2018. A 50% increase was reported in hospitalization, emergency, and intensive care units during the same time period.

**Conclusions:**

The program addressed a gap in the provision of PPC to children in Cali. It shows effective strategies used to implement a PPC program and how the referral times, coordination of care, communication with other hospital services were improved while providing compassionate/holistic care to children with life-limiting and threatening diseases and in end-of-life. The implementation of this program has required the onset of specific strategies and arrangements to promote awareness and education proving it a hard task, yet not impossible.

**Supplementary information:**

**Supplementary information** accompanies this paper at 10.1186/s12904-020-00674-2.

## Background

The prevalence of complex life-threatening diseases in children has increased significantly worldwide [1, 2]. In the United Kingdom, the prevalence of limiting and life-threatening diseases increased from 12 per 10,000 population in 2003 to 16 per 10,000 by 2007 [3, 4]. The prevalence of these diseases in Latin America is still to be determined since there is still a lack of information. The World Health Organization (WHO) stated that such conditions could benefit from the palliative care (PC) approach [5]. In this light, the burden of these diseases and thus the need for PC becomes a growing necessity for healthcare systems worldwide and a moral imperative [6].

A recent regional study assessing the development of pediatric palliative care (PPC) in Europe estimated that approximately 150,000 children need PPC every year [7]. The region offers a total of 680 services to address the need, of which 133 are hospices, 385 are home-care services and 162 are hospital services [7]. The statistics about PC need are expected to be larger in Latin America due to the health inequalities that the region faces. The Lancet Commission on Palliative Care reported an unaddressed lack of access to PC and pain relief worldwide which mainly affects low- and middle-income countries (LMICs) where the provision for PPC and pain relief are estimated to be neglected [8]. A systematic review reported that 66.7% of the countries in South America did not have any PPC activity until 2011, when the first integrated PPC service was documented [9]. The field of PC has continued to develop and become an active area of research and advocacy. Even though a new definition of the concept is available, the generalized provision of healthcare in Latin America has focused on improving access to specialized adult services [10]. This results in an untrained health workforce in the field of pediatrics and a lack of PPC services to address the current need [11].

The 2012 Atlas of Palliative Care in Latin America reported Colombia as having four hospice-type residencies, only one second-level service, and 13 tertiary care units that serve both adults and children [12]. There are still no available data regarding the national need for PC; however, according to the Childhood Cancer Outcomes Surveillance System (VIGICANCER), the incident rate of pediatric cancer from 1977 to 2011 is comparable with affluent countries [13].

To date, national PC services are estimated to have grown almost 500% in the past 5 years. However, developing PPC in Colombia requires overcoming even greater barriers than for adults, mainly due to the absence of PC academic training programs for pediatricians and the lack of related educational objectives for other healthcare professionals [9, 14].

In light of the pressing need to promote PPC in Latin America and considering the characteristics of PPC in Colombia based on the recommendations from the *American Academy of Pediatrics* (AAP), the *Institute of Medicine* (IoM), and the *International Meeting for Palliative Care in Children’s Trento* (IMPaCCT) for the introduction of PPC teams [12, 15, 16], the objective of this paper is to report the process of implementing the PPC program *“Taking Care* of You” (*TCY*) in the city of Cali in Colombia. As with other LMIC health inequalities and a lack of scientific literature on the subject, Cali represents a context where promoting PPC might seem to be an impossible task. This article reflects on the strategies attempted for developing a multidisciplinary program that could provide coordinated care and symptom management to children with life-threatening and limiting conditions, geared toward reducing suffering and improving patients and families’ quality of life.

## Contextual factors

### Geographic and demographic context

Colombia has a population of 48,258,494 inhabitants, 51.2% of the population is female, and 22.6% are between 0 and 14 years of age [17]. Cali is a city with 2.5 million inhabitants [18]. There is limited data available regarding the number of pediatric patients with a need for PC or PPC services available in the country; however, the age-standardized annual incidence rate of childhood cancer in Cali from 1977 to 2011 was 141 cases per million [13, 19].

### Colombian legal framework for PC regulation

Since 2014, the country has passed relevant measures related to PPC regulation. Important milestones include the regulation of services in the country, including those aimed at the pediatric population [20], guidelines for inclusive care in PC, differentiating attention for adults and children [21], specific standards for Children Cancer Care Units (CCCU) where PC and pain treatment must be guaranteed from the beginning of treatment [22], a cancer control plan with a mention of PC [23], and regulating the process of dignifying death for children and adolescents, allowing euthanasia [24]. (Colombian regulatory framework is summarized in Appendix 1 under supplementary data).

### The program “Taking Care of You”

The program operates in the Fundación Valle del Lili, a nonprofit, teaching hospital that functions as a referral center for the southwestern region of Colombia. It has 177 pediatric beds and cares for approximately 50,000 children annually.

Our institution has a general PC program that has operated since 2007 and is led by family medicine physicians with a multidisciplinary team. The general PC program had served the entire population including children until late 2017, when the specialized PC program for pediatric patients called Taking Care of You (*TCY)* was launched. TCY is led by a pediatrician with a team that includes a family medicine physician, a nurse, a social worker, and a psychologist.

The main objective of the program is to provide coordinated, multidisciplinary and humanized care to promote quality of life for patients under the age of 18 with life-threatening and limiting conditions in any hospital care service [emergency room, hospital room, pediatric intensive care unit (PICU), neonatal intensive care unit (NICU), and outpatient setting]. Patients are treated through an inter-consultation or first-time assessment by the multidisciplinary PPC team.

This multidisciplinary team is designed to provide coordination of care, support in clinical decision-making, facilitated communication, pain and other symptom management, advanced care planning, end of life care, and bereavement follow up [25], among others that will be described later.

### Program strategies

To fulfill the baseline program goals and objectives, an eight-step strategy was implemented. This included education and awareness in PPC (a question-based strategy, summarized in Appendix 2 under supplementary data), institutional support, the participation of the PPC team in academic and healthcare activities, advocacy with other actors in the healthcare system, capacity building in PPC, the formation of a multidisciplinary team led by a pediatrician with training in PPC, and research that is described in Table 1.

**Table 1:** Eight-step implementation strategy

Strategies	Objective	Setting	Deliverable
**Education**	Inform healthcare providers, families, health care professionals in order to raise awareness	Hospitalization, emergency, PICU, NICU, CCU	Educational discussion meetings, conferences for caregivers, patients, and families.
**Institutional Support**	To establish collaborative support between board directors/decision-makers and PPC program leader	Board of directors Education committee Management committee	Reports and meetings that showed the enhancement of humanized care, improvement in patient experience and healthcare personnel experience, health outcomes, and finally resource optimization.
**Academic Support**	To educate HCW in PPC and its impact in the healthcare services	All HCW from hospitalization, PICU, NICU, CCU, BMTU	Meetings Conferences Workshops Educational material
**Advocacy With Other Actors In The Health System**	To promote PPC with healthcare authorities, scientific societies, the academic community, and stakeholders	health insurance, scientific associations, patient associations, national palliative care scientific associations, territorial health entities	Boards Meetings Conferences, Diplomacy
**Capacity Building**	To train and educate the TCY program team in PPC	Postgraduate education: -Universidad Internacional de La Rioja Continued education: -Harvard Medical School courses	Master’s degree Palliative care education and practice course
**Multidisciplinary Team**	To organize a multidisciplinary team in PPC that guarantees a comprehensive and holistic approach for the patient and their family needs	FVL	Inclusion of different healthcare workers including a psychologist, social worker, nurse, and spiritual counselor
**Specialized Pediatric Team Leader**	To guarantee a comprehensive approach to the pediatric medical conditions in PC	FVL, Pediatrician leader, Department of Palliative Care	A physician with specific training in pediatrics and PPC
**Research**	To create an information system that characterizes the population and identifies clinical, economic, and social problems that may contribute to solving scientific gaps and support multilevel decision-making.	FVL	PPC research group Support of research assistant

*BMTU* Bone Marrow Transplant Unit, *HCW* Healthcare Workers, *PPC* Pediatric Palliative Care, *CCU* Children Cancer Units, *PICU* Pediatric Intensive Care Unit, *NICU* Neonatal Intensive Care Unit, *TCY* “Taking Care of You”, *FVL* Fundacion Valle del Lili

## Methods

Two approaches were followed to describe the outcomes of the program. First, a categorization of the strategies was conducted to assist with its implementation, and second, descriptive statistics were used to describe the program’s outcomes.

### Mapping implementation strategies

A theory-based method was applied to describe the implementation process of our program. The approach was based on the Poot et al. article [26] where a matrix was retrospectively developed from descriptive frameworks. To analyze and display the strategies implemented, a matrix exercise was performed.

#### Strategies of the program *TCY* in the matrix

The strategies were categorized retrospectively with the Cochrane Effective Practice and Organization of Care (EPOC) [27, 28], Review Group Taxonomy 2015 [28], and grouped into the categories and subcategories: Implementation Strategies, Financial arrangements and Delivery arrangements Table 2.

**Table 2:** Effective Practice and Organisation of Care (EPOC) Taxonomy “Implementation Strategies” and “Financial Arrangements” Fitting the “Taking Care of You” (TCY) Program Strategy and Objectives

Subcategory	TCY strategy	Strategy objective
**Implementation Strategies** Interventions designed to bring about changes in healthcare organizations, the behavior of healthcare professionals or the use of health services by healthcare recipients		
**Category: Interventions targeted at healthcare workers**		
**A. Communities of Practice**	Local advocacy to convene capacity building	Train a specialized PPC team through graduate programs abroad
**B. Educational Materials**	Design and create written, and online evidence-based information material	Supply healthcare professionals with key objective topics and information on PPC
**C. Educational Meetings**	Local, and national educational courses and workshops	Create a successful method to favor mass training and raising awareness on PPC approach and principles for healthcare professionals
**D. Interprofessional Education**	Coach national multidisciplinary courses and participation in postgraduate university courses	Increase national multidisciplinary knowledge on palliative philosophy
**E. Patient-Mediated Interventions**	Medical, psychological and social work evaluation of the patient and family to discuss as part of multidisciplinary medical board meetings	Provide a psychosocial and medical perspective of the patient and family prior to multidisciplinary decision-making meetings
**Financial Arrangements** Changes in how funds are collected, insurance schemes, how services are purchased, and the use of targeted financial incentives or disincentives		
**Category: Collection of funds**		
**F. External Funding**	Apply for funding through a research grant	Promote and sustain pediatric palliative care in a middle-income country
**Category: Insurance schemes**		
**G. Community-Based Health Insurance**	Held Advocacy Reunions with health care providers locally	Lower access barriers for patients and families of MIC
**Category: Mechanisms for the payment of health services**		
**H. Payment Methods for Health Workers**	Reunions with the board of directors and decision-makers emphasizing the added value of PPC, based on enhancing patient and family satisfaction, patient experience, health humanization, and resource optimization	Obtain institutional support to consolidate the team and decrease the access barrier
**Delivery Arrangements** Changes in how, when, and where healthcare is organized and delivered, and who delivers healthcare.		
**Category: Where care is provided and changes to the healthcare environment**		
**I. Site of service delivery**	Promote patient attention in the outpatient scenario through medical order	Since most of the patients are referred to the program from hospitalization, we make sure they can continue attention in the outpatient ward
**Category: Who provides care and how the healthcare workforce is managed**		
**J. Role expansion or task shifting**	Coached local interdisciplinary team meetings, educational meetings among the general PC group.	Guide the conformation of the Pediatric Palliative Care team
**Category: Coordination of care and management of care processes**		
**K. Care pathways**	Held institutional multidisciplinary meetings with local health care providers	Contextualize life-limiting-and-threatening disease
**L. Case management**	Participated in multidisciplinary board meetings with treating specialist and several homecare services	Coordinate and guarantee an integrative followup to improve patients care
**M. Communication between providers**	Coached local interdisciplinary team meetings, support for clinical improvement plans of the team and regional educational meetings	Facilitating and establishing communication and developing an improved dialogue.
**N. Continuity of care**	While in hospitalization we hold medical board meetings with interdisciplinary teams and promote continuity through outpatient setting followup	Ensuring the responsibility of care and bereavement followup
**O. Disease management**	Coached educational team meetings, regional meetings with health care professionals and healthcare providers	Promote adequate quality of life during the health-disease-attention process
**P. Patient-initiated appointment systems**	Providing phone advisory 24 h 7 days a week	Around-the-clock availability for care consultation to direction the family and bereavement care
**Q. Referral systems**	Coached educational sessions with the hospital’s pediatric departments	Educating about the importance of involving comprehensive care and patients with complex chronic diseases who are candidates for referral to the PPC team
**R. Shared decision-making**	Meetings and constant communication is held with TCYteam, treating specialist and the family	Establish individualized management goals
**S. Teams**	Coached local interdisciplinary team meetings, support for clinical improvement plans, and regional educational meetings	Establishing a multidisciplinary team that provides organizational status, coordinated care, and capability based on individualized relevance and effectivity
**T. Transition of Care**	Interdisciplinary meetings between treating specialist, our team and the family	Provide objective information to the family when a patient’s treatment changes from curative to palliative

*PPC* Pediatric Palliative Care, *MIC* Middle Income Country, *TCY* “Taking Care of You”, *MIC* Middle-Income countries

The matrix combines two frameworks: first, levels of organization influenced by the implementation [29], and second, the domains of implementation [30] to provide a comprehensive matrix where defined project activities can be positioned according to their intended target domain and level, facilitating a structured description of the project [26, 29]. The matrix displays the implementation strategies, Financial arrangements, and delivery arrangements. Different sources of information were cross-referenced in a database to improve the quality of the information.

### Program outcomes

A descriptive analysis of the variables was performed using the program’s administrative registry, no clinical chart review took place, nonetheless, this study was submitted to the Institutional Research Board (IRB) and Ethical Committee. The results were reported using measures of central tendency and dispersion, according to data distribution. Categorical variables were summarized as percentages. The variables analyzed included a detailed analysis of the children served and the referral service, type of pathologies (i.e., oncologic vs nononcologic), and place of death per year. All analyses were performed using Microsoft Excel 2016. Information from referral motive was gathered as part of an internal administrative process through an online survey (Appendix 3 provided in supplementary data). No literature search took place, but it did undergo IRB and Ethical Committee review.

### Education strategies

There were continuous educational activities in the pediatric units that emphasized on the importance and health benefits from the early referral of children with limiting and life-threatening conditions to PPC, advancing to the progressive growth of the program. Educational activities include PPC and pain management workshops, communication role play games, printed materials, PPC subjects in the grand rounds, clinical case discussions, among others.

## Results

### Coverage

**Fig. 1:**
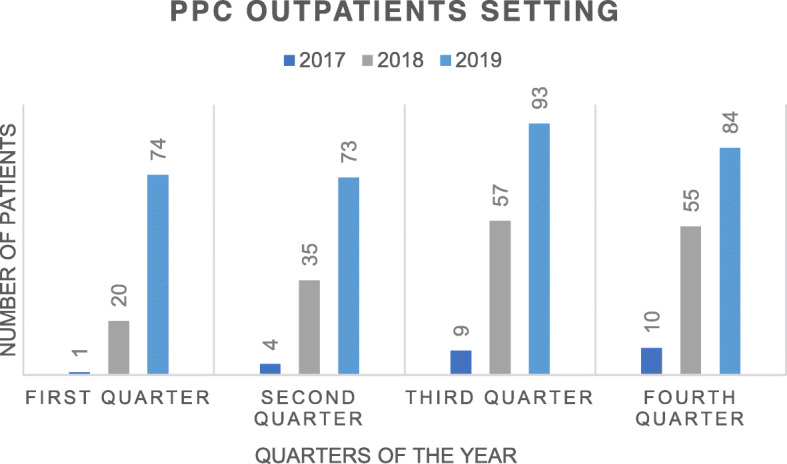
Comparison of the number of patients assessed by the PPC program in the outpatient setting between 2017 and 2019. PPC Pediatric Palliative Care

**Fig. 2:**
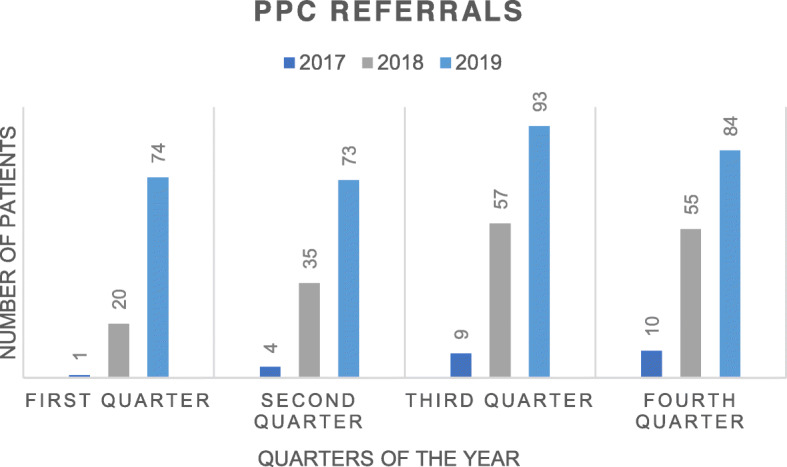
Comparison of the number of Inpatients referred to the program from 2017 to 2019. PPC Pediatric Palliative Care

Since the beginning of the program in 2017, a total of 1965 children have been referred. In 2017, before the specific PPC program was created, 146 pediatric patients were assessed by the general PC team, whereas in 2019, 1 year after the start of the “Taking Care of You” program, 771 children were assessed in the pediatric inpatient services and 324 new patients were assessed in the outpatient setting of the hospital. This represents an 81 and 93% increase PPC demand in the inpatient and outpatient services, respectively (see Figs. 1 and 2).

A relevant aspect in the program development was making a strategic alliance with the PICU and the pediatric oncology unit, which favored early integration of the PPC team in the unit’s activities and allowed support in decision-making to promote patient quality of life. This also allows continuity of care, interdisciplinary communication, and therapeutic adherence.

### Disease prevalence at the moment of referral to the program

Patients referred to the program were classified as oncological and non-oncological. We found a similar proportion in both categories, including inpatient and outpatient care. In our institution, the most frequent oncological clinical conditions were central nervous system tumors (53%) (mainly medulloblastoma and low- and high-grade gliomas), followed by bone tumors (26%) (Osteosarcoma and Ewing’s sarcoma). On the other hand, non-oncological conditions were mainly related to severe neurological compromise in 38% of the cases (including congenital malformations and chromosomal abnormalities, such as trisomy’s, microcephaly, cerebral palsy), followed by inherited muscle disorders and rare diseases.

### Cause of referral

The main causes for referral in outpatient and inpatient setting was support during therapeutic withholding and withdrawal of a treatment that was already established (89%), followed by end-of-life care (86%), Communication support (70%), Pain and symptom control (69%), Psychosocial support (67%), Decision-making support (63%), and Health Care coordination (57%). The most frequent reasons for referral to the PPC program by departments are summarized in Table 3, the majority came from the pediatric inpatient ward (52%) and the PICU (23%). The median time from referral to death was 12 days, with an interquartile range of 2 to 61 days.

**Table 3:** Pediatric Patient Referral by Departments

Pediatric Patient Referral by Departments								
Department	2017		2018		2019		Total	
	n	%	n	%	n	%	n	%
General pediatric ward	4	**40.0%**	398	**50.0%**	634	**54.5%**	1036	**52.7%**
Pediatric ICU	–	–	192	**24.3%**	266	**22.8%**	458	**23%**
Pediatric Emergency room	5	**50.0%**	126	**15.6%**	150	**12.7%**	281	**14.3%**
Neonatal ICU	1	**10.0%**	61	**7.7%**	87	**7.5%**	149	**7.6%**
Bone Marrow Transplant	–	–	13	**1.6%**	28	**2.4%**	41	**2.1%**
Total general	10	**100%**	790	**100%**	1165	**100%**	1965	**100%**

*ICU* Intensive Care Unit

### Place of death and bereavement follow-up

The place of death of most of the patients was the hospital Table 4. Eleven percent of the relatives attended the bereavement workshops in the 2017–2018 time period and 25% in the 2018–2019 time period. The *TCY* program accompanied bereavement of parents and families through, follow-up calls, sending condolence letters, and bereavement support groups, with a symbolic butterfly release in addition to psychological interventions.

**Table 4:** Place of death and bereavement workshop attendance comparison per year

Place of death	Total ***n*** = 288	2017^a^ ***n*** = 57	2018^b^ ***n*** = 111	2019 ***n*** = 120
Emergency	24	2	11	11
General Pediatric ward	215	39	83	93
Another healthcare institution	14	3	1	10
Home	35	13	16	6
**Attendance to bereavement workshop**	79	6	12	61

^a^General PC program

^b^Start of the PPC program

The program has had around-the-clock availability, it received 81 phone calls in 2018 and 244 phone calls in 2019, related to symptom management (58%) and care coordination (42%).

### Strategies

**Fig. 3:**
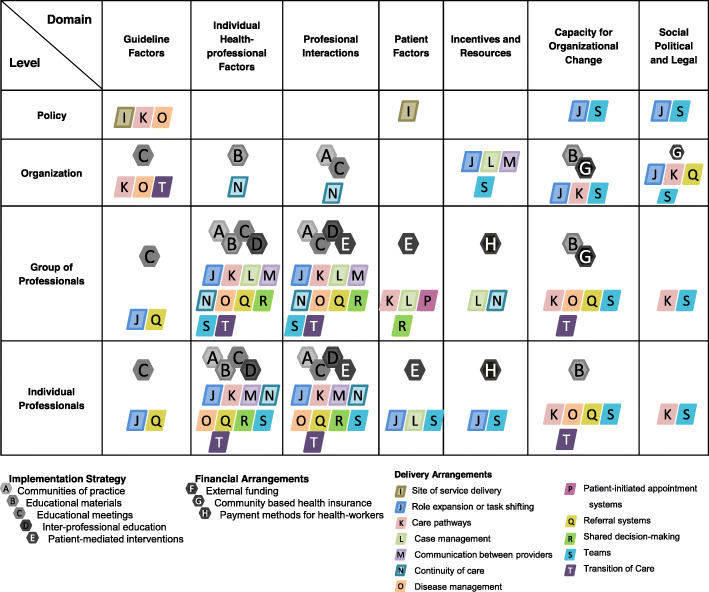
Integration of the EPOC categories “Implementation Strategy”, “Financial Arrangements ”, “Delivery Arrangements” in the matrix of targeted “levels of organization” and “Domains of implementation”. Note EPOC: Effective Practice and Organization of Care

Figure 3 shows the integration of the EPOC categories on a single matrix displaying the categorization for the domains: implementation strategies, financial arrangements, and delivery arrangements. The program has focused on advancing in educational and professional interaction, through promoting communication between interdisciplinary teams. Second, it aimed on developing organizational change by task shifting, promoting incentives and resources and pursuing change in social, political and legal frameworks. The program has not stressed change at a policy level, though small changes have been reported at generating specific roles for the program, determining the site of services and providing disease management specifically from a PPC perspective.

## Discussion

The program has focused on an unaddressed gap, the provision of PPC for children in Cali. It has improved referral times, coordination of care, availability of compassionate/holistic care to children with limiting and life-threatening diseases, and end-of-life care. The implementation of this program has required the onset of specific strategies and arrangements to promote awareness and education, which has been a difficult task. With this implementation experience, we intend to contribute to the PC philosophy in pediatric patients’ care and hope it serves as a model for other regions and countries.

Establishing a PC team with skills, training, and exclusive activity assistance for the pediatric population was a crucial element to expand PPC demand for children with complex chronic diseases and to increase the program demand a three-fold compared with the previous year. As described in the Children’s Hospital of Eastern Ontario, Ottawa, a significant increase in patient referrals was associated with the inclusion of social work resources and experienced PC physicians [31]. Additionally, Education was key for the progressive growth of the program [32] and could be a strategy to lower resistance from healthcare professionals, since this has been described to be a barrier [33].

When using the theory-based method description applied to our TCY program implementation, we recognized the program has focused on advancing individual professional and groups of professionals in their capacities and competencies and on generating organizational change to find a niche within the hospital without influencing policy. Small changes at the policy level can be accounted for by the definition of specific PPC roles and settings for patient care.

Disease prevalence at the moment of referral to the program displayed the most frequent diagnoses in oncological and non-oncological groups were neurological, similar to other implementation studies carried out worldwide [34]. We corroborate that children with neurological conditions, even if not progressive, experience high distress due to symptom control difficulties [32, 35], the high burden associated with care [36, 37], and potential complications that could cause premature death [32, 34], pointing out the important role a PC team plays in the multidisciplinary management of these patients.

Referral causes regarding our PPC program were associated with supporting decisions around withholding or withdrawing treatment, and end-of-life care, especially in the NICU and the PICU services, with an average time from referral until death of 12 days. This underlines late integration of PPC in the wide and varied conditions susceptible to the service. In the largest retrospective medical record review for a PPC population in the US by Thrane et al., they evidenced that the time from referral to death was 4.4 months [38]. Highlighting children are still not being referred to PC until mere days before death, not in line with recommendations from the WHO and other organizations [39] that recommend PPC to be provided after diagnosis of life-threatening diseases, which in turn could result in less aggressive care and better outcomes at the end-of-life [40].

The global trend is to promote death at home with patient-family comfort and support measures [41]; however, in pediatrics, multiple difficulties arise when trying to promote this measure, and the hospital scenario is the most frequent place of death [42]. Our team recognized the same difficulty, and most of our patients (74%) died in the hospital. Some of the barriers identified in favoring end-of-life care at home were related to fear from parents or caregivers to face the death of their child alone, the high attention needs of a patient at the end-of-life, the lack of home care programs with PC training, and the absence of pediatric hospices in the country, similar to what is described in the literature [41–43]. However, some families were able to care for their children at home during their final days.

Finally, family support during bereavement is an essential biopsychosocial need for the family’s well-being after the loss of a child [44]. Our program does follow-up to support parents in grief: however, bereavement workshop attendance has been low (25%). We consider it an important aspect to improve by increasing coverage and offering more family services. Future goals, achievable through My Child Matters grant, include improving inclusion and access by benefitting more than 500 children in the region, accompanying 1000 families and relatives, and educating more than 1500 healthcare professionals. We also seek to make the enlarged program sustainable.

### Limitations

This study shows the implementation of a PPC program in a teaching, general hospital in Cali, Colombia. This is a descriptive study, and the data were retrospectively collected, which implies that selection and information bias could be introduced during its development. To mitigate this situation, our databases were crossed with different sources of institutional information to improve the quality of information. On the other hand, our institution is a specialized reference center for patients with highly complex diseases; therefore, the reported number of patients who benefit from the program is high when compared with other institutions. Additionally, the Colombian healthcare system is based on universal coverage provided by several insurance companies, and as such, patients are often transferred to other institutions due to individual insurance preferences or administrative limitations, making it hard to keep track of all PPC patients. Likewise, there was no guarantee that all the patients with cancer and life-threatening conditions received outpatient care and completed follow-up through the *TCY* program because patients often need difficult-to-obtain authorization from their health entity to receive specialized care, such as PPC.

Despite these limitations, our implementation model could be an example to mitigate gaps in the PPC service for Latin America and the Caribbean, where availability and access to a PPC team are scarce.

## Conclusions

The creation of a specialized PPC service increases patient referrals and favors a comprehensive approach to patients with life-threatening and limiting conditions in a general hospital. Institutional support, philanthropic support, awareness, and education are essential for the viability and impact of PPC programs. There are still many opportunities for improvement, such as the referral time and death of patients, the creation of a pediatric hospice, and improving the bereaved follow up, among others. The implementation of a PPC program in Colombia is a difficult but not impossible task to accomplish. With this paper, we hope that other PPC programs can be strengthened or new ones can be created in Latin America and the Caribbean.

## Supplementary information

**Additional file 1: Appendix 1.** Colombian legal framework related to palliative care. Provide a summary of the Colombian laws and legislations that support Pediatric Palliative Care and promote quality of life for children with life-limiting and threatening diseases.

**Additional file 2: Appendix 2.** Program approach strategy. The team developed a question-based strategy to address institutional education and awareness in PPC.

**Additional file 3: Appendix 3.** Institutional Satisfaction Survey. Online institutional questionnaire applied to healthcare professionals through the pediatric departments, to quantify satisfaction and referral motives.

## Data Availability

The datasets used and/or analyzed during the current study are available from the corresponding author on reasonable request.

## Abbreviations

TCY: *“Taking Care of You”*
PPC: Pediatric Palliative Care
PC: Palliative Care
AAP: *American Academy of Pediatrics*
IoM: The *Institute of Medicine*
IMPaCCT: *International Meeting for Palliative Care in Children’s Trento*
CCCU: Children Cancer Care Units
CCU: Children Cancer Units
*NHPCO*: *National Hospice and Palliative Care Organization*
PICU: Pediatric intensive care unit
NICU: Neonatal intensive care unit
ASOCUPAC: *Asociacion de Cuidados Paliativos de Colombia*
WHO: The World Health Organization
HIV: Human immunodeficiency virus

## References

[1] Bogetz JF, Ullrich CK, Berry JG. Pediatric hospital care for children with life-threatening illness and the role of palliative care. Pediatr Clin North Am. 2014;61(4):719-33. 10.1016/j.pcl.2014.05.002. 10.1016/j.pcl.2014.05.002 25084720

[2] Downing J, Birtar D, Chambers L, Drake R, Gelb B, Kiman R (2012). Children’s palliative care: a global concern. Int J Palliat Nurs.

[3] Fraser LK, Miller M, Hain R, Norman P, Aldridge J, McKinney PA (2012). Rising national prevalence of life-limiting conditions in children in England. Pediatrics.

[4] Together for Short Lives. Making and impact: moments that count [Internet]. 2018–2019. [cited 2020 Apr 22]. Available from: https://www.togetherforshortlives.org.uk/about-us/making-an-impact/impact-reports/.

[5] World Health Organization (WHO) (2018). Integrating Palliative Care and Symptom Relief into Pediatrics: A WHO guide for health planners, implementers and managers.

[6] Stevenson M, Achille M, Lugasi T (2013). Pediatric palliative care in Canada and the United States: A qualitative metasummary of the needs of patients and families. J Palliat Med.

[7] Arias-Casais N, Garralda E, Rhee JY, de Lima L, Pons JJ, Clark D (2019). EAPC Atlas of Palliative Care in Europe 2019.

[8] Knaul FM, Farmer PE, Krakauer EL, De Lima L, Bhadelia A, Jiang Kwete X (2018). Alleviating the access abyss in palliative care and pain relief—an imperative of universal health coverage: the lancet commission report. Lancet..

[9] Florez SP, Tovar MB, Leon MX, Villegas K, del DP V, Granados CE (2015). Caracterización del conocimiento en cuidado paliativo pediátrico y percepción debarreras por parte de los pediatras y residentes de pediatría. Med Paliativa.

[10] Radbruch L, de Lima L, Knaul F, Wenk R, Ali Z, Bhatnaghar S, et al. Redefining Palliative Care – a New Consensus-based Definition. J Pain Symptom Manage. 2020; Available from. 10.1016/j.jpainsymman.2020.04.027. 10.1016/j.jpainsymman.2020.04.027 PMC8096724 32387576

[11] Observatorio Colombiano de Cuidados Paliativos, Universidad El Bosque (2016). Anuario del Observatorio Colombiano De Cuidados Paliativos.

[12] Families I of M (US) C on P and E-LC for C and T, Field MJ, Behrman RE. In: Field MJ, Behrman RE, editors. Institute of Medicine (US) Committee on Palliative and End-of-Life Care for Children and Their Families, When Children Die: Improving Palliative and End-of-Life Care for Children and Their Families: National Academies Press; 2003. [cited 2020 Mar 9]. Available from: https://www.ncbi.nlm.nih.gov/pubmed/25057608. 25057608

[13] Bravo LE, García LS, Collazos P, Aristizabal P, Ramirez O (2013). Descriptive epidemiology of childhood cancer in Cali, Colombia 1977-2011. Colomb Med.

[14] Ospina ML, Huertas JA, Montaño JI, Rivillas JC. Observatorio Nacional de Cáncer Colombia. Rev Fac Nac Salud Pública. 2015;33(2) [cited 2019 Nov 25]. Available from. 10.17533/udea.rfnsp.v33n2a.

[15] Nelson RM, Botkin JR, Kodish ED, Levetown M, Truman JT, Wilfond BS (2000). Palliative care for children. Pediatrics.

[16] Craig F, Abu-Saad Huijer H, Benini F, Kuttner L, Wood C, Feraris PC (2008). IMPaCCT: Standards pädiatrischer Palliativversorgung in Europa. Der Schmerz.

[17] Censo Nacional de Población y Vivienda 2018 [Internet]. [cited 2020 Mar 9]. Available from: https://www.dane.gov.co/index.php/estadisticas-por-tema/demografia-y-poblacion/censo-nacional-de-poblacion-y-vivenda-2018.

[18] Departamento Administrativo Nacional de Estadística. Proyecciones de población municipales por área. Censo general 2005 [Internet]. 2005 [cited 2020 Mar 9]. Available from: https://www.dane.gov.co/index.php/estadisticas-por-tema/demografia-y-poblacion/censo-general-2005-1.

[19] Ramirez O, Aristizabal P, Zaidi A, Gagnepain-Lacheteau A, Ribeiro RC, Bravo LE (2018). Childhood cancer survival disparities in a universalized health system in Cali, Colombia. Pediatr Hematol Oncol J.

[20] El Congreso de Colombia. Ley No 1733 Consuelo Devis Saavedra. Bogota D.C; 2014. [cited 2020 Feb 24]. Available from: http://wsp.presidencia.gov.co/Normativa/Leyes/Documents/LEY%1733%DEL%08%DE%SEPTIEMBRE%DE%2014.pdf.

[21] Ministerio de Salud y Protección Social (2014). Ley 1733 (septiembre 8).

[22] Ministerio de Salud y Protección Social. Resolución 1477 (abril 22). Bogotá D.C; 2016. p. 1–30. [cited 2020 Mar 9]. Available from: https://www.minsalud.gov.co/Normatividad_Nuevo/Resolución-1477%de%2016.pdf.

[23] Ministerio de Salud y Protección Social, Instituto Nacional de Cancerología E.S.E. Plan Nacional para el control del cáncer en Colombia, 2012–2021. Bogotá D.C; 2012. p. 1–85. [cited 2020 Mar 9]. Available from: https://www.minsalud.gov.co/sites/rid/Lists/BibliotecaDigital/RIDE/IA/INCA/plan-nacional-control-cancer-2012-2020.pdf.

[24] Ministerio de Salud y Protección Social. Resolución 825 (marzo 9). Bogotá D.C; 2018. p. 1–16. [cited 2020 Mar 9]. Available from: https://www.minsalud.gov.co/sites/rid/Lists/BibliotecaDigital/RIDE/DE/DIJ/resolucion-825-de-2018.pdf.

[25] Verberne L, Kars M, Schepers S, Schouten-van Meeteren A, Grootenhuis M, Delden J (2018). Barriers and facilitators to the implementation of a paediatric palliative care team. BMC Palliat Care.

[26] Poot AJ, de Waard CS, Wind AW, Caljouw MAA, Gussekloo J (2017). A structured process description of a pragmatic implementation project: Improving integrated care for older persons in residential care homes. Inq (United States).

[27] Proctor EK, Powell BJ, McMillen JC (2013). Implementation strategies: recommendations for specifying and reporting. Implement Sci.

[28] Arrangements D (2015). EPOC Taxonomy – topics list.

[29] Flottorp SA, Oxman AD, Krause J, Musila NR, Wensing M, Godycki-Cwirko M (2013). A checklist for identifying determinants of practice: A systematic review and synthesis of frameworks and taxonomies of factors that prevent or enable improvements in healthcare professional practice. Implement Sci.

[30] Shortell SM (2004). Increasing value: a research agenda for addressing the managerial and organizational challenges facing health care delivery in the United States. Med Care Res Rev.

[31] Vadeboncoeur CM, Splinter WM, Rattray M, Johnston DL, Coulombe L (2010). A paediatric palliative care programme in development: trends in referral and location of death. Arch Dis Child.

[32] van Dyck PC, Kogan MD, McPherson MG, Weissman GR, Newacheck PW (2004). Prevalence and characteristics of children with special health care needs. Arch Pediatr Adolesc Med.

[33] Keele L, Keenan HT, Sheetz J, Bratton SL (2013). Differences in characteristics of dying children who receive and do not receive palliative care. Pediatrics..

[34] Hubble RA, Ward-Smith P, Christenson K, Hutto CJ, Korphage RM, Hubble CL (2009). Implementation of a palliative care team in a pediatric hospital. J Pediatr Health Care.

[35] Kiman R, Varela R (2011). Establishing a pediatric palliative care team in an Argentinian hospital. Eur J Palliat Care.

[36] Lyons-Warren A (2018). Update on palliative Care for Pediatric Neurology. Am J Hosp Palliat Med.

[37] Lyons-Warren AM, Stowe RC, Emrick L, Jarrell JA (2019). Early Identification of Pediatric Neurology Patients With Palliative Care Needs: A Pilot Study. Am J Hosp Palliat Med.

[38] Thrane SE, Maurer SH, Cohen SM, May C, Sereika SM (2017). Pediatric palliative care: a five-year retrospective chart review study. J Palliat Med.

[39] National Consensus Project for Quality Palliative Care (2018). Clinical Practice Guidelines for Quality Palliative Care 4 th edition Publisher: National Coalition for Hospice and Palliative Care Clinical Practice Guidelines for Quality Palliative Care, 4 th edition.

[40] Cuervo-Suarez MI, Claros-Hulbert A, Manzano-Nunez R, Muñoz M, García X (2020). Pediatric Palliative Care During End of Life: A Privilege of a Few in a Tertiary Referral Hospital From Colombia. Am J Hosp Palliat Med.

[41] Villanueva G, Murphy MS, Vickers D, Harrop E, Dworzynski K (2016). End of life care for infants, children and young people with life limiting conditions: summary of NICE guidance. BMJ..

[42] Widger K, Seow H, Rapoport A, Chalifoux M, Tanuseputro P (2017). Children’s end-of-life health care use and cost. Pediatrics.

[43] Gao W, Verne J, Peacock J, Stiller C, Wells C, Greenough A (2016). Place of death in children and young people with cancer and implications for end of life care: a population-based study in England, 1993-2014. BMC Cancer.

[44] Donovan LA, Wakefield CE, Russell V, Cohn RJ (2015). Hospital-based bereavement services following the death of a child: a mixed study review. Palliat Med.

