# Supplementary material for: Advancing pediatric palliative care in a low-middle income country: an implementation study, a challenging but not impossible task
Source: BMC Palliat Care. 2020 Nov 6;19:170. doi: 10.1186/s12904-020-00674-2 (PMC7648318; doi:10.1186/s12904-020-00674-2)
Supplement: Supplementary file 1 — Additional file 1: Appendix 1. Colombian legal framework related to palliative care. Provide a summary of the Colombian laws and legislations that support Pediatric Palliative Care and promote quality of life for children with life-limiting and threatening diseases. [file 12904_2020_674_MOESM1_ESM.docx]

**Supplementary document:**

**Appendix 1.** Colombian legal framework related to palliative care

| **Framework** | **Year** | **Description** |
| --- | --- | --- |
| Law 1384  *“Ley Sandra Ceballos”* | 2010 | Specifically supports the diffusion and guarantees palliative care assistance |
| Resolution 1841 | 2013 | Ten-Year Public Health Plan 2012- 2021 |
| Resolution 1383 | 2013 | Ten-Year Cancer Control Plan 2012 -2021 |
| Law 1733  *“Ley Consuelo Deivis”* | 2014 | Regulate palliative care services for comprehensive management of patients with terminal, chronic, degenerative, and irreversible diseases in any phase of the disease ensuring quality of life. |
| Law 1753 | 2015 | The base for the issue of the National Development Plan. Strengthens human resource capacities of health providers that attend older adults, relating social health determinants, rights focus, life course, primary health care (palliative care, home care, and caregiver care) |
| Resolution 429 | 2016 | Regulates the comprehensive health care policy |
| Circular 022 | 2016 | Guidelines that manage access to opioid medication and pain management |
| Circular 023 | 2016 | Instructions to guarantee palliative care rights for requiring patients |
| Resolution 1477 | 2016 | Childhood Cancer Care Unit (CCCU) |
| Resolution 825 | 2018 | Right for children and adolescents to die with dignity |
