# Supplementary material for: Advancing pediatric palliative care in a low-middle income country: an implementation study, a challenging but not impossible task
Source: BMC Palliat Care. 2020 Nov 6;19:170. doi: 10.1186/s12904-020-00674-2 (PMC7648318; doi:10.1186/s12904-020-00674-2)
Supplement: Supplementary file 2 — Additional file 2: Appendix 2. Program approach strategy. The team developed a question-based strategy to address institutional education and awareness in PPC. [file 12904_2020_674_MOESM2_ESM.docx]

**Appendix 2.** Program approach strategy

| **Guiding Questions** | **Action Field** |
| --- | --- |
| What is the *TCY*  program? | Publicize the new PPC program, its objectives, scope, mission, vision, and activities to develop |
| Why was the *TCY* program created? | Educating who is a candidate for follow-up by the PPC team and awareness of the importance involving comprehensive care and patients with complex chronic diseases. This aspect is of vital importance to guarantee sustainability, growth and the program’s impact |
| The existence of the Program *TCY* , what for? | Providing comprehensive care to children and adolescents with chronic, life-threatening and limiting conditions to promote adequate quality of life during the health-disease-attention process. Coordinated care that includes physical, psychological and spiritual needs of the patients and their families |
| Whom is this program aiming at? | The program aims to treat patients 18 years old or less with life-limiting and threatening diseases |

*TCY “Taking Care of You”,* PPC pediatric palliative care program
