# Supplementary material for: Advancing pediatric palliative care in a low-middle income country: an implementation study, a challenging but not impossible task
Source: BMC Palliat Care. 2020 Nov 6;19:170. doi: 10.1186/s12904-020-00674-2 (PMC7648318; doi:10.1186/s12904-020-00674-2)
Supplement: Supplementary file 3 — Additional file 3: Appendix 3. Institutional Satisfaction Survey. Online institutional questionnaire applied to healthcare professionals through the pediatric departments, to quantify satisfaction and referral motives. [file 12904_2020_674_MOESM3_ESM.docx]

**Additional File 3. Appendix 3**

**Institutional Satisfaction Survey**

1. **Gender**
   1. Female
   2. Male

1. **Profession**
   1. Nurse
   2. Medical Physician
   3. Psychologist
2. **Institucional role (you can pick more than one option)**
   1. Academic
   2. Clinical- assistance
   3. Administrative
   4. other
3. **Which pediatric clinical department do you belong to?**
   1. Emergency department
   2. Hospitalization department
   3. Oncology department
   4. Intensive care Unit
   5. Neonatal Intensive Care Unit
   6. Outward setting
4. **Select all the contribution the pediatric palliative care team has provided your department (you can pick more than one option)**
   1. Multidisciplinary team coordination
   2. Pain control and other symptoms treatment recomendations
   3. Decision-making support
   4. Facilitate patient and family communication
   5. Psycho-social support
   6. Care Humanization
5. **What is your perception regarding patient and family care, provided by the pediatric palliative care team? (pick one option)**
   1. Very useful
   2. Useful
   3. Neutral
   4. Useless
   5. Not useful at all
6. **When do you request Pediatric Palliative Care evaluation (you may check more than one option):**
   1. Support during therapeutic effort limitation
   2. End-of-life care
   3. Communication support
   4. Pain and Symptom control
   5. Psychosocial support
   6. Decision-making support
   7. Health care coordination
7. **Do you consider the pediatric palliative care program favors caregiver well-being?**
   1. Yes
   2. No
